# Frailty syndrome and socioeconomic and health characteristics among older adults

**DOI:** 10.25100/cm.v48i3.1978

**Published:** 2017-09-30

**Authors:** Darlene Mara dos Santos Tavares, Thais Aline de Freitas Corrêa, Flavia Aparecida Dias, Pollyana Cristina dos Santos Ferreira, Maycon Sousa Pegorari

**Affiliations:** 1 Department of Nursing in Education and Community Health, Nursing Graduate Program, Universidade Federal do Triângulo Mineiro. Minas Gerais, Brazil; 2 Universidade Federal do Amapá. Macapá, Amapá, Brazil.

**Keywords:** frail elderly, aging, health status, socioeconomic factors, health services for the aged, activities of daily living, ancianos frágiles, envejecimiento, estado de salud, factores socioeconómicos, salud del anciano, actividades cotidianas, functional disability for basic and instrumental activities of daily life

## Abstract

**Objective:**

To investigate the association of frailty syndrome with socioeconomic and health variables among older adults.

**Methods:**

This is a cross-sectional, observational and analytical household research conducted with a sample of 1,609 urban elderly. We used: semi-structured questionnaire, scales (Katz, Lawton and shortened version of Geriatric Depression Scale) and Fragility Phenotype proposed by Fried. Descriptive analysis was performed along with a bivariate and multinomial logistic regression model (*p* <0.05).

**Results:**

The prevalence of pre-frailty condition was 52.0% and the fragility corresponded to 13.6%. Pre-frailty and frailty associated factors were, respectively: age range between 70-79 years and ≥80 years; one to four morbidities and five or more morbidities categories; functional disability for basic and instrumental activities of daily life and depression indicative; whilst lack of a companion or income and female gender were only associated to pre-frailty.

**Conclusion:**

The conditions of pre-frailty and frailty levels were elevated with negative effects on the health of the elderly.

## Resumen

**Objetivo:**

Investigar la asociación del síndrome de fragilidad con variables socioeconómicas y de salud de los adultos mayores.

**Métodos:**

encuesta domiciliaria transversal, observacional y analítico realizado con una muestra de 1,609 personas mayores urbano. Fueron utilizados: cuestionario semi-estructurado, escalas (Katz, Lawton y depresión geriátrica acortado) y Fragilidad Fried fenotipo. Se realizaron los siguientes análisis: descriptivo, bivariado y modelo de regresión logística multinomial (*p* <0.05).

**Resultados:**

La prevalencia de la condición pre-fragilidad fue del 52.0% y la fragilidad correspondió a 13.6%. Los factores asociados a la prefragilidad y fragilidad fueron, respectivamente: a los intervalos de edad de 70-79 años y ≥80 años; una a cuatro morbilidades y cinco o más categorías de morbilidad; incapacidad funcional para actividades básicas e instrumentales de la vida diaria y indicativo de depresión; en cuanto que la ausencia de compañero y ingreso y el género femenino se asociaron a la prefragilidad.

**Conclusión:**

Las condiciones de los niveles previos a la fragilidad y la fragilidad fueron elevados con efectos negativos sobre la salud de los ancianos.

## Introduction

The frailty is a clinical condition associated with pathological aging and biological vulnerability ^1^. This syndrome results from the decrease in physiological reserves and deregulation of multiple systems ^2^ ^,^ ^3^. 

In this context, we emphasize that studies in Spain ^4^ ^,^ ^5^ and in Brazil ^6^ ^,^ ^7^ with older people in the community have identified increase of pre-frail followed by frail individuals. 

The scientific literature has reported that some socioeconomic conditions are associated with the presence of this syndrome. National research have shown that among community elderly, frailty relates to older age, being single and low education ^7^ ^,^ ^8^; it is highlighted that sex shows mixed results in these studies ^7^ ^,^ ^8^. However, a study in Spain has not identified differences in the prevalence of frailty in relation to sex, marital status and education ^4^; it is stressed that this study was conducted among older adults aged 65 years and older including institutionalized and community individuals of rural and urban areas ^4^. These data highlight the need for studies to deepen the knowledge about this topic.

Furthermore, greater chances for adverse health conditions are associated with frailty in this population ^6^. Research that integrates multi-center study on the Frailty in Brazilian Elderly (*Rede FIBRA*) conducted in Brazil, with community seniors aged 65 years and over showed that the frailty has been associated with functional dependence in basic, instrumental and advanced activities of the daily life as well as depressive symptoms ^6^. Frailty is associated with comorbidities not only in studies with Brazilian elderly ^6^ ^,^ ^9^ but also, for example, in those resident in Spain and Taiwan ^5^ ^,^ ^10^. It is noteworthy that these studies have been conducted with seniors aged 65 years and over; however, in Brazil, individuals aged 60 years or older are considered elderly ^11^.

Considering this gap related to age and the lack of studies in this country, which is justified by the lack of proper tools and professionals who are able to correctly identify the frail elderly ^12^, this research aims to contribute to expand knowledge about the socioeconomic and health factors related to frailty among older adults aged 60 years and over in the community. 

Thus, the aim of this study was to investigate the association of the frailty syndrome with socioeconomic and health variables among older adults.

## Material and Methods

### Design and sample

This study refers to a household, observational study, with quantitative, transversal and analytical approach, developed in 2012 with elderly residents in urban areas in the city of Uberaba, Minas Gerais, Brazil. It is part of a larger research that comprises a longitudinal study, developed by the *Grupo de Pesquisa em Saúde Coletiva* (Research Group on Public Health) of the Federal University of Triangulo Mineiro (UFTM).

For sample composition, we obtained a list with the Zoonosis Center of the city, containing the name and address of the elderly. For selection of the elderly we considered 95% confidence, 80% test power, margin of error of 4.0% for the interval estimates and an estimated ratio of π = 0.5 for the proportion of interest. Thus, 2,118 elderly composed the sample of the larger project in 2012, of which 1,693 were interviewed and 265 were canceled due to death and 160 due to cognitive decline.

Inclusion criteria for this study were individuals aged 60 years or older, living an urban area, not presenting cognitive decline, being able to walk, being allowed the use of assistive devices for gait (cane, crutch or walker), agreeing to participate after signing the Informed Consent Form (ICF) and have fully answered the questions involving the items of frailty phenotype. The elderly were excluded if they presented inability to walk alone, severe sequelae of stroke, Parkinson's disease in severe or unstable condition/terminal stage, severe vision and/or hearing deficit (29); and those who did not answer all frailty items (55). Therefore, 1,609 elderly participated in this study.

### Procedures

For data collection we selected 10 interviewers, who were trained by the main researcher on how to approach and conduct the interview with the elderly and on the ethical aspects related to the research. The interviews were reviewed by supervisors who found incomplete fields or inconsistency in the responses. When it was necessary, the questionnaire was returned to the interviewer, who contacted the elderly for proper filling. 

### Tools

To collect data we used the Mini Mental State Examination (MMSE), translated and validated in Brazil ^13^, to verify whether the elderly had cognitive decline. The MMSE has a total score ranging from 0 to 30 points, and the cutoff points for cognitive decline in the elderly consider the level of education: 13 points for illiterate, 18 points for 1 to 11 years of education and 26 points for more than 11 years of education ^13^.

To characterize the sociodemographic data and identification of comorbidities we used an instrument built by the Research Group on Public Health. 

To evaluate the functional capacity we used the index of Independence in Activities of Daily Living (Katz index) adapted to the Brazilian reality ^14^. This scale consists of six items that measure the performance of a person in self-care activities, i.e., they assess the performance of basic activities of daily living (BADL) ^14^. It was also used the Lawton and Brody Scale (1969) adapted in Brazil ^15^, which is composed of nine items assessing instrumental activities of daily living (IADL), with scores ranging from 7 to 21 points ^15^. 

The presence of indicative of depression was measured from the shortened version of Geriatric Depression Scale, validated in Brazil ^16^. This scale consists of 15 objective questions with a total score of 15 points. It is considered positive screening for depression when the elderly get 6 points or more on the scale ^16^.

The fragility syndrome was identified by means of the five items described as components of frailty phenotype proposed by Fried *et al*. ^2^, as follows: (1) unintentional loss of weight evaluated by the following question: "In the last year, have you lost more than 4.5 kg or 5% of body weight unintentionally (i.e., without diet or exercise)?"; (2) self-reported exhaustion and/or fatigue, assessed by two questions (items 7 and 20) of the Brazilian version of the depression scale of the Center for Epidemiologic Scale (CES-D). Seniors who had a score 2 or 3 in any of the items met the criterion of fragility to this item ^17^; (3) decrease in muscle strength, verified based on the grip strength, through the manual hydraulic dynamometer, JAMAR type, model SAEHAN® SH5001 - 973. Three measurements were obtained, presented in kg/force (kgf), with an interval of one minute between them, and the average value of these measurements was considered, adopting the cutoffs proposed by Fried *et al*. ^2^; (4) delay in walking speed, in which we considered the time (in seconds) taken to walk a distance of 4.6 m. The elder covered a total distance of 8.6 m: the first 2 m and 2 final m were not considered in calculating the time spent on the march. Three measurements were performed, presented in seconds, considering the average of them. It was used as standard a professional stopwatch Vollo® brand, model VL-1809 and the cutoff points proposed by Fried *et al*. ^2^ and (5) low level of physical activity, verified by the long version of the International Physical Activity Questionnaire (IPAQ) adapted for seniors ^18^. The classification used for this component followed the recommendations of the American College of Sports Medicine and the American Heart Association, which consider active those who do physical activity for 150 min or more per week; and inactive the seniors who do physical activity from zero to 149 min weekly ^19^. Elderly patients with three or more of these items have been classified as frail, and those with one or two items, as pre-frail and those with all negative tests, robust or non-frail ^2^.

### Variables

We included in this study the following variables: 

Socio-demographic and economic variables: gender (male and female), age range in years (60-69, 70-79 and ≥80 years), marital status (with or without a companion), literacy in years of study (none, one to four years and five or more years), individual income in minimum wages (none, one or less, two or three, four or more minimum wages); 

Self-reported morbidities: rheumatism, arthritis/osteoarthritis, osteoporosis, asthma/bronchitis, tuberculosis, embolism, high blood pressure (hypertension), poor circulation, heart problems, diabetes, obesity, stroke, Parkinson's, urinary incontinence, fecal incontinence, constipation, trouble sleeping, cataracts, glaucoma, spinal problems, kidney problems, sequel accident/trauma, malignant tumors, benign tumors, vision problems, others; 

Number of morbidities: none, one to four and five or more morbidities; 

Functional capacity in BADL: bathing, dressing, toileting, transferring, sphincter control, food; 

BADL functional incapacity: yes or no; 

Functional capacity in IADL: using the telephone, traveling, shopping, preparing meals, performing housework, medication use and handling money; 

IADL functional incapacity: yes or no; 

Presence of indicative of depression: yes or no; 

Fragility syndrome rating: non-frail, pre-frail, frail. 

### Data analysis

After data collection it was built an electronic database in Microsoft Office Excel® 2007 program, processed in a personal computer, by two people, in double entry. The existence of duplicate records and the consistency of the fields were verified, by checking the wrong typing. When there was inconsistency in data, the original interview was retaken for correction. Later, the database was imported into the software "Statistical Package for Social Sciences" (SPSS) version 17.0 for data analysis. 

Descriptive statistical analysis was performed for categorical variables, from absolute and percentage frequencies. In order to verify socioeconomic and health variables association with pre-frailty and frailty condition, a bivariate preliminary analysis was conducted, using the chi-squared test. Tests were considered significant when *p* <0.10.

The interest variables, according to the established inclusion criteria (*p* <0.10), were included in the multivariate regression model. Frailty and pre-frailty associated factors were identified by multivariate analysis with prevalence odds ratio estimative. Thus a multinomial logistic regression model (saturated model) was used, considering a significance level of 5% (*p* <0.05 and a confidence interval (CI) of 95%. 

### Ethical considerations

The project was submitted to the Ethics Committee in Research with Human Beings of UFTM and approved under the opinion No. 2265. Interviewers approached the elderly participants at home, showed the ICF and after clarifying the doubts of the participants, asked them to sign the aforementioned form and started the interview.

## Results

The prevalence of frail elderly was 13.6% (n= 219), whereas pre-fragility corresponded to 52.0% (n= 836).

Regarding the associated factors, the preliminary bivariate analysis variables, which were submitted to the multivariate analysis, according to the established inclusion criteria (*p* <0.10), were: gender, age range, marital status, literacy, individual monthly income, morbidities, BADL and IADL functional incapacity and depression indicative (Tables 1 and ^2^).

**Table 1:** Elder's socio-demographic and economic characteristics according to the frailty status. Uberaba, Minas Gerais, Brazil, 2012.

Variables	Non frail		Pre-frail		Frail		χ^2^	p*
	n	%	n	%	n	%		
Gender								
Male	212	38.3	304	36.4	57	26.0	10.683	0.005
Female	342	61.7	532	63.6	162	74.0		
Age range (years)								
60-69	279	50.4	284	34.0	48	21.9	126.632	<0.001
70-79	221	39.9	404	48.3	84	38.4		
≥80	54	9.7	148	17.7	87	39.7		
Marital status								
Without a companion	284	51.3	506	60.6	131	59.8	12.530	0.002
With a companion	270	48.7	329	39.4	88	40.2		
Literacy (years)								
0	91	16.5	182	21.8	57	26.0	21.882	<0.001
1-4	278	50.3	441	52.8	119	54.3		
≥5	184	33.3	213	25.5	43	19.6		
Individual monthly income**								
0	34	6.1	58	7.0	16	7.3	27.331	<0.001
<1	237	42.9	434	52.0	131	59.8		
2-3	212	38.3	268	32.1	60	27.4		
≥4	70	12.7	74	8.9	12	5.5		

*p <0.05;

**Minimum wage in the period: R$ 622.00.

**Table 2:** Elder's health characteristics according to frailty status. Uberaba, Minas Gerais, Brazil, 2012.

Variables	Non-frail		Pre-frail		Frail		χ^2^	p*
	n	%	n	%	n	%		
Morbidities								
0	33	6.0	23	2.8	1	0.5	91.375	<0.001
1-4	303	54.7	325	38.9	58	26.5		
≥ 5	218	39.4	488	58.4	160	73.1		
BADL functional incapacity								
Yes	55	9.9	185	22.1	90	41.1	96.323	<0.001
No	499	90.1	651	77.9	129	58.9		
IADL functional incapacity								
Yes	278	50.2	572	68.4	202	92.2	129.756	<0.001
No	276	49.8	264	31.6	17	7.8		
Depression indicative								
Yes	86	15.5	216	25.8	116	53.0	114.458	<0.001
No	468	84.5	620	74.2	103	47.0		

*p= <0.05; BADL: Basic activities of daily living; IADL: Instrumental activities of daily living.

Among the reported morbidities in both groups, there was prevalence of hypertension, back and vision problems, with the highest percentage in frail, followed by pre-frail and non-frail elderly. 

Regarding the functional disabilities to perform BADL, the highest dependency rates were to continence in the three groups.

Frail elderly had greater dependency percentage to traveling, housework and shopping compared to pre-frail and non-frail seniors.

Included variables in the multivariate model of the multinomial logistic regression are presented in Table 3. Were confirmed as frailty and pre-frailty associated factors, respectively: age range of 70-79 years and ≥80 years; one to four morbidities and five or more morbidities categories; BADL and IADL functional incapacity and depression indicative. Moreover, lack of a companion or monthly income and female gender were associated to pre-frailty (Table 3).

**Table 3:** Final model of multinominal logistic regression using frailty and pré-frailty associated variables. Uberaba, Minas Gerais, Brazil, 2012.

Variables	Pre-frails			Frails		
	OR	CI 95%	p*	OR	CI 95%	p*
Age range (years)						
60-69	1			1		
70-79	1.68	1.31-2.16	0.001	2.13	1.40-3.31	0.001
≥80	2.19	1.50-3.29	0.001	7.40	4.40-12.46	0.001
Gender						
Male	1			1		
Female	1.38	1.06-1.80	0.017	0.86	0.56-1.33	0.500
Marital status						
Without companion	1.56	1.21-2.01	0.001	1.09	0.73-1.63	0.676
With a companion	1			1		
Literacy (years)						
0	0.97	0.67-1.40	0.968	1.11	0.63-1.95	0.718
1-4	0.99	0.72-1.36	0.978	1.10	0.67-1.66	0.802
≥5	1			1		
Income (wages)†						
0	1.96	1.06-3.62	0.031	1.96	0.70-5.48	0.197
<1	1.34	0.88-2.03	0.173	1.77	0.83-3.77	0.139
2-3	1.03	0.68-1.55	0.888	1.31	0.61-2.82	0.483
≥4	1			1		
Morbidities						
0	1			1		
1-4	1.41	0.79-2.53	0.249	4.28	0.52-35.08	0.176
≥5	2.43	1.34-4.41	0.003	9.16	1.12-74.89	0.039
BADL functional incapacity						
Yes	1.87	1.33-2.63	0.001	3.06	1.98-4.73	0.001
No	1			1		
IADL functional incapacity						
Yes	1.68	1.32-2.13	0.001	6.36	3.67-11.02	0.001
No	1			1		
Depression indicative						
Yes	1.44	1.07-1.94	0.016	4.05	2.72-6.02	0.001
No	1			1		

OR: *Odds Ratio*; CI: confidence interval, *p= <0.05;

1: reference category;

†minimum wage in 2012 in Brazil (R$ 622.00);

BADL: Basic activities of daily living;

IADL: Instrumental activities of daily living.

## Discussion

Research in Brazil ^6^ ^-^ ^8^ that addressed the frailty syndrome showed lower results compared to the present study, although in the municipality of Santa Cruz-RN ^20^ higher percentages were observed for the prevalence of pre-frailty (60.1%) and frailty (17.1%). Variations in prevalence of this condition may be related to methodological approaches in the measurements of the components of frailty phenotype and also to the influence of socioeconomic and health characteristics from the population context observed.

Consistent results with this study have been verified in research of *Rede FIBRA* - Campinas-SP, whose highest percentage of frail seniors occurred among older elderly ^8^. Another research in Bahia had also observed an association between frailty and age range; the older the elder, the higher frailty and prefrailty probability (*p* <0.001) ^21^. Older elderly are more vulnerable to stressful events, have higher functional overload and difficulties in maintaining homeostasis ^3^, suggesting that the higher the chronological age, the greater the tendency of becoming frail elderly ^20^ ^,^ ^22^. 

Frail elderly are at higher risk for health and require increased demand for care, resulting in overload for both families and for the health system. It is therefore important that the family is inserted in the care of the frail elderly, providing necessary support to the individual and to the healthcare team. Investment in measures to prevent the fragility ensures better quality of life and lower cost to health services. The demand for long term care and social support reflect the need for these seniors to be public policy targets ^23^.

With regard to gender, the results are corroborated by a national research, which presented a more frequent pre-frailty condition in female gender ^8^ and differed of an international survey ^24^ showing an absence of this association. This result may be related to the fact that women suffer greater loss of physiological reserve compared to men ^25^ and are more likely to worse socioeconomic and health conditions, which may reflect in higher chances of fragility ^26^. In addition, there is also the longer life expectancy and lower muscle mass indexes of women ^27^, which may favor pre-frailty condition when compared to men. 

When referring to marital status, a study conducted in Brazil ^6^ corroborates with the present survey, since there was a larger pre-frail elders proportion without a companion when compared to non-frail.

Concerning income, divergent results were evidenced in a study conducted in Santa Cruz-RN ^20^, where there was no association between elderly frailty conditions and income. The association between aspects such as low socioeconomic status and frailty in the elderly may be result of accumulated disadvantages throughout the life course, added to age-related deficits ^8^. 

With regard to morbidities, the results of this study confirm the scientific literature ^2^ ^-^ ^3^ ^,^ ^9^ ^-^ ^10^ ^,^ ^20^. Similarly to this study, hypertension stood out among the Taiwanese community elderly, and it was self-reported by 49.5% in frail and 47.7% in pre-frail seniors ^10^. Other research has shown that individuals with frailty syndrome have higher blood pressure and other cardiovascular risk factors, lower HDL and increased abdominal fat when compared to non-fragile elderly ^28^. Frailty may potentiate the development or progression of chronic diseases, probably due to low levels of activity or due to other mechanisms that interfere with homeostasis in the elderly ^3^. On the other hand, the presence of concomitant illnesses may contribute to the development of frailty in elderly ^29^, association identified in a Belo Horizonte survey, which observed frail elders presented greater chance of presenting a higher number of comorbidities (OR= 1.27) ^6^. 

With regard to the functional disabilities to perform BADL, study showed that 70.7% of elderly patients with urinary incontinence (UI) presented at least three criteria indicative of fragility and their occurrence was higher in frail seniors, which corroborates the findings of the present investigation ^30^. The UI in frail elderly have negative repercussions, making it a syndromic condition that presents several risk factors that interact, such as the changes inherent to age and the comorbidities ^31^, which suggests the need for research and implementation of actions aimed at the reduction or prevention of this inability associated with the frailty syndrome. Among the strategies to minimize the occurrence of UI, there is highlight to guidance for the elderly in relation to food, fluid intake and practices that strengthen the pelvic floor muscles. Early measures to prevent the UI and the frailty condition in the elderly can have a positive impact in these individuals' lives, reducing the economic, psychological and social burden ^30^.

The results observed for the association between frailty and dependence for performance of BADL and IADL corroborate Brazilian ^6^ ^,^ ^20^ and American studies ^2^ ^,^ ^3^. Research conducted in Ribeirão Preto-SP showed that the higher the level of fragility, the greater the level of dependency in the elderly ^12^. So the difficulty in performing basic and instrumental activities of daily living may cause the increased dependence and emergence of frailty syndrome ^32^.

Relating to disabilities for IADL, research investigating the association between social support and fragility found a significant association between this syndrome and help with household chores ^9^, diverging from the current study that identified dependency to domestic work, traveling and shopping. The frailty syndrome is closely related to the disability, requiring the incorporation of preventive measures in order to mitigate and slow functional decline in the elderly, with a view to promote active aging ^6^.

Similarly to the present investigation, in a study conducted with elders from Belo Horizonte-MG ^6^, the presence of depression indicative was associated with frailty and pre-frailty conditions (*p*<0.001). In the same direction, a literature review identified depressive symptoms as antecedents that may favour frailty syndrome development in elders ^33^.

Knowing more deeply the relationship between fragility and depression may contribute to the clarification of the factors that influence the etiology and prognosis of the syndrome, favoring the development of specific actions and the integration of different geriatric fields ^34^.

The limitations of this study refer to the use of the questionnaire for the self-report of morbidities and the cross-sectional design that hinders the advancement in time analysis, because of reverse causality bias, not allowing finding causal relationship.

## Conclusion

Frail elderly accounted for 13.6%, while the pre-fragile seniors corresponded to 52.0%. Frailty and pre-frailty associated factors were, respectively: age range of 70-79 years and ≥80 years; one to four morbidities and five or more morbidities categories; BADL and IADL functional incapacity and depression indicative. Moreover, lack of a companion or monthly income and female gender were associated to pre-frailty. 

These data highlight the need for health actions focused on the socioeconomic characteristics of the elderly. Thus, preventive measures can be established considering the higher association of frailty syndrome with socioeconomic aspects.

In addition, screening for frailty may contribute to the establishment of preventive measures in its evolution. There is also the need for monitoring the health conditions of these elderly aiming to postpone the appearance of comorbidities, including depression, and their control in order to help favoring them to minimize the development of this syndrome. Activities focused on functionality can also help in this regard.
