# Supplementary material for: Frailty syndrome and socioeconomic and health characteristics among older adults
Source: Colomb Med (Cali). 2017 Sep 30;48(3):126–31. doi: 10.25100/cm.v48i3.1978 (PMC5687864; doi:10.25100/cm.v48i3.1978)
Supplement: Translation

# Translation: Síndrome de fragilidad y características socioeconómicas y de salud de los adultos mayores

## Introducción

La fragilidad es una condición clínica asociada con el envejecimiento patológico y vulnerabilidad biológica ^1^. Este síndrome es resultado de la disminución de las reservas fisiológicas y desregulación de múltiples sistemas ^2^^,^^3^. En este contexto, se enfatiza que estudios con adultos mayores que viven en la comunidad, realizados en España ^4^^,^^5^ y Brasil ^6^^,^^7^ identificaron un incremento de la proporción de los participantes considerados pre-frágiles y frágiles.

La literatura científica sobre el tema indica que algunas condiciones socioeconómicas están asociadas a la presencia de este síndrome. Los adultos mayores que viven en la comunidad, el síndrome de fragilidad estuvo relacionado con los considerados más viejos, solteros y con una escolaridad baja ^7^^,^^8^; destacándose que la variable sexo presentó resultados contradictorios al ser analizados conjuntamente con la fragilidad ^7^^,^^8^. Sin embargo, un estudio español no identificó diferencias entre la prevalencia del síndrome de fragilidad relacionado al sexo, estado civil y educación ^4^; se enfatiza que este estudio fue realizado con adultos mayores con edad >65 años, y que su muestra incluyó adultos mayores que vivían en la comunidad y en asilos de las zonas urbanas y rurales ^4^, destacando la necesidad de la realización de nuevos estudios con el intuito de profundizar el conocimiento en este tema.

Asimismo, se encuentran mayores posibilidades de condiciones adversas a la salud asociadas con la fragilidad en esta población ^6^. En el estudio multicentrico de Fragilidad en Adultos mayores Brasileños (Red FIBRA), con participantes que viven en la comunidad mayores de 65 años se identificó que la fragilidad está asociada con la dependencia funcional para las actividades básicas, actividades instrumentales y actividades avanzadas de la vida diaria y la presencia de síntomas depresivos ^6^. La asociación entre fragilidad y las comorbilidades no solo es evidenciada en estudios brasileños ^6^^,^^9^, si no también, en estudios realizados con adultos mayores de España y Taiwan ^5^^,^^10^. Se resalta que estos estudios fueron realizados con adultos mayores con 65 años y más, pero que en el Brasil, personas con 60 años y más son considerados adultos mayores ^12^.

Teniendo en cuenta esta brecha de la edad y la ausencia de estudios en el Brasil justificado por la falta de herramientas adecuadas para la identificación de los adultos mayores frágiles ^12^, este estudio tuvo como objetivo investigar la asociación entre el síndrome de fragilidad con las variables socioeconómicas y de salud de los adultos mayores, con ≥60 años que viven en la comunidad

## Material y Métodos

### Diseño y muestra

Estudio cuantitativo, de corte transversal y analítico desarrollado en el año 2012 con adultos mayores que viven en el área urbana de la ciudad de Uberaba, Minas Gerias, Brasil. Esta investigación forma parte de un estudio longitudinal desarrollado por el *Grupo de Pesquisa em Saúde Coletiva* (Grupo de Investigación en Salud Pública) de la Universidad Federal del Triángulo Minero (UFTM).

La muestra se obtuvo de la relación del Centro de Zoonosis de la ciudad, el cual tenía los nombres y direcciones de los adultos mayores. Para la selección de la muestra fue considerado un nivel de confianza del 95% y una potencia de la prueba del 80%, con un margen de error del 4.0% para estimar los intervalos y una proporción estimada de π= 0.5 para las proporciones de interés. La muestra final del proyecto mayor estuvo compuesta de 2,118 adultos mayores de los cuales 1,693 fueron entrevistados y 265 eliminados debido al fallecimiento y 160 por presentar un déficit cognitivo.

Los criterios de inclusión fueron individuos con edad ≥60 años, vivir en el área urbana, no presentar déficit cognitivo, capaz de movilizarse o uso de tecnologías de asistencia para deambular (bastón, muleta o andador), aceptar en participar posteriormente a la firma del Consentimiento Informado y de haber respondido todos los ítems del cuestionario sobre el fenotipo de fragilidad. Los adultos mayores fueron excluidos si presentaron incapacidad para caminar solos, secuelas severas de Accidente Cerebral Vascular, Parkinson o una condición inestable/estado terminal, disminución de la visión y/o audición (29) y aquellos que no respondieron todos los ítems de fragilidad (55), por lo tanto, la muestra final estuvo conformada por 1,609 adultos mayores.

### Procedimiento

La recolección de los datos fue realizada por 10 entrevistadores que fueron entrenados por la investigadora principal dando énfasis de cómo debe ser abordara y conducida la entrevista y los aspectos éticos de la investigación. Las encuestas fueron revisadas por los supervisores. Cuando se encontraron datos incompletos o respuestas inconsistentes, el cuestionario fue devuelto al entrevistador, para que completara o corrigiera la información junto con el adulto mayor.

### Instrumentos

Para la recolección de los datos fue utilizado el cuestionario Mini Examen del Estado Mental (MEEM), traducido y validado para el Brasil ^13^, con la finalidad de identificar el déficit cognitivo en el adulto mayor. El MEEM presenta un escore de 0 a 30 puntos, y el punto de corte de la escala además considera el nivel educativo del adulto mayor. 

Para considerarlo con déficit cognitivo es utilizado los siguientes puntos de corte: 13 puntos para analfabetos, 18 para aquellos que tienen ente 1 a 11 años de estudios y 26 con más de 11 años de estudios ^13^.

Para la caracterización sociodemográfica e identificación de comorbilidades fue utilizado el instrumento construido por el grupo de investigación en salud pública.

En la evaluación de la capacidad funcional fue utilizado el índice de Independencia para las Actividades de la Vida Diaria (Índice de Katz) adaptado para la población brasileña ^14^. La escala está conformada por seis ítems que identifican el desempeño de las actividades de autocuidado, estas evalúan el desempeño de las Actividades Básicas de la Vida Diaria (ABVD) ^14^. Asimismo, fue utilizado la Escala de Lawton y Brody (1969) adaptado para el Brasil ^15^, compuesto de nueve ítems que evalúan las Actividades Instrumentales de la Vida Diaria (AIVD) con un escore de 7 a 21 puntos.

Para la identificación de síntomas depresivos fue utilizada la versión corta de la Escala de Depresión Geriátrica validada para el Brasil ^16^. La escala contiene 15 preguntas objetivas con un escore de 15 puntos y se considera la presencia de estos síntomas cuando el adulto mayor obtiene 6 puntos o más.

El síndrome de fragilidad fue identificado por medio de los cinco componentes del fenotipo de fragilidad propuesto por Fried *et al*. ^2^: (1) pérdida involuntaria de peso evaluada por la siguiente pregunta: ¿en el último año, usted perdió más de 4.5 kg o 5% de su peso corporal de forma involuntaria (es decir, sin la realización de dieta o ejercicios)?; (2) reporte de cansancio y/o fatiga evaluado por dos preguntas (ítems 7 y 20) de la versión brasileña de la escala de Depresión del Center for Epidemiologic Scale (CES-D). Adultos mayores que tengan 2 o 3 puntos en cualquiera de los ítems cumplirán con el criterio de fragilidad ^17^; (3) disminución de la fuerza muscular verificado en base a la fuerza de prensión de la mano, a través del dinamómetro hidráulico manual tipo JAMAR, modelo SAEHAN® SH5001-973. Tres medidas fueron obtenidas evaluadas en kg/fuerza (kgf), con un intervalo de un minuto entre estas y la media de estas medidas fue considerada adoptando el punto de corte propuesto por Fried *et al*. ^2^, (4) disminución de la velocidad de la marcha considerando el tiempo (segundos) y recorriendo una distancia de 4.6 m., para esto el adulto mayor camino una distancia de 8.6 m. el cual los dos metros iniciales y finales no fueron considerados para el cálculo del tiempo de la marcha. Tres medidas en segundos fueron realizadas y fue considerado una media de estas, para este control fue utilizado el cronómetro profesional marca Vollo®, modelo VL-1809 y utilizado los puntos de corte propuesto por Fried *et al*. ^2^, y (5) disminución de la actividad física, evaluado por la versión del International Physical Activity Questionnaire (IPAQ) adaptado para adultos mayores ^18^. La clasificación utilizada para este componente fue realizada por medio de las recomendaciones del American College of Sports Medicine y del American Heart Association, el cual considera al adulto mayor activo si realiza actividad física de 150 min o más por semana e inactivo para aquellos que realizaban actividades de 0 a 149 min por semana ^19^. Aquellos adultos mayores con tres o más de estos componentes fueron clasificados como frágiles, uno o dos componentes como pré-frágiles y sin ningún componente considerado robustos o no frágiles ^2^.

### Variables

En el estudio fueron incluidas las siguientes variables:

Variables sociodemográficas y económicas: sexo (masculino y femenino), intervalo de edad (60-69, 70-79 y ≥80 años), estado civil (con y sin pareja), escolaridad en los años de estudio (sin estudios, uno a cuatro años y cinco años y más), ingresos económicos sueldos mínimos (sin sueldo, uno o menos, dos o tres, cuatro y más);

Declaración de morbilidades: reumatismo, artritis/osteoartritis, osteoporosis, asma/bronquitis; tuberculosis, embolismo, hipertensión arterial, problemas de circulación, problemas cardiacos, diabetes, obesidad, Accidente Cerebro Vascular, Parkinson, incontinencia urinaria, incontinencia fecal, constipación, problemas para dormir, cataratas, glaucoma, problemas de columna, problemas renales, secuelas de accidente/trauma, tumores malignos, tumores benignos, problemas de visón, otros;

Numero de morbilidad: ninguna, una a cuatro y cinco y más;

Capacidad funcional para las ABVD: baño, vestido, uso del retrete, movilidad, continencia y alimentación;

Incapacidad funcional para las ABVD: si o no;

Capacidad funcional para las AIVD: uso del teléfono, transporte, hacer compras, preparar alimentos, realizar actividades domésticas, uso de medicación y manejo del dinero;

Incapacidad funcional para las AIVD: si o no;

Presencia de síntomas depresivos: si o no;

Síndrome de fragilidad: no frágil pre-frágil y frágil.

### Análisis de los datos

Después de la recolección de los datos fue construido un base de datos electrónica en el programa Microsoft Office Excel® 2007, procesado por dos personas con doble entrada. La existencia de duplicación de los datos y la consistencia de estos fue verificada por medio de la doble digitación. Cuando se verificaban inconsistencias, se realizó la corrección respectiva consultando el instrumento de recolección de datos. Posteriormente, los datos fueron importados al programa estadístico "Statistical Package for Social Sciences" (SPSS) versión 17.0.

Se realizó un análisis descriptivo para las variables categóricas y frecuencia y porcentajes para los valores absolutos. Se verificó la asociación entre las variables socioeconómicas y de salud con la condición pre-frágil y frágil, el análisis preliminar bivariado fue realizado utilizando la prueba de qui-cuadrado. Para todas las pruebas fue considerado significante cuando *p* <0.10.

Las variables de interés, de acuerdo a criterios de inclusión establecidos (*p* <0.10), fueron incluidas en el modelo de análisis de regresión multivariada. Los factores asociados con las categorías frágil y pre-frágil fueron identificados por el análisis multivariado con estimación del Odds Ratio. Posteriormente, fue utilizado el modelo de regresión logística multinomial (modelo de saturación), considerando un nivel de significancia de 5% (*p* <0.05) y un intervalo de confianza (IC) del 95%.

### Consideraciones éticas

El proyecto fue sometido al Comité de Ética e Investigación con seres Humanos de la UFTM y aprobado según el proceso n^o^ 2265. Los entrevistadores agendaron visitas en el domicilio del adulto mayor donde era firmado el Consentimiento Informado posterior a la aclaración por dudas de los participantes y poder dar inicio a las entrevistas.

## Resultados

La prevalencia de fragilidad en el adulto mayor fue de 13.6% (n= 219), sin embargo 52.0% fueron considerad pre-frágiles (n=836).

En relación a los factores asociados, para con el analisis preliminar bivariado, se incluyeron al análisis multivariado de acuerdo a los criterios de inclusión (*p* <0.10) las variables: sexo, intervalo de la edad, el estado civil, la escolaridad, el ingreso mensual, las morbilidades. Incapacidad funcional para las ABVD y AIVD y síntomas depresivos (Tablas 1 y ^2^).

**Tabla 1:** Características sociodemográficas y económicas de los adultos mayores de acuerdo al estado de fragilidad. Uberaba, Minas Gerais, Brasil, 2012.

Variables	No frágil		Pre-frágil		Frágil		χ^2^	p*
	n	%	n	%	n	%		
Sexo								
Masculino	212	38.3	304	36.4	57	26.0	10.683	0.005
Femenino	342	61.7	532	63.6	162	74.0		
Intervalo de edad (años)								
60-69	279	50.4	284	34.0	48	21.9	126.632	<0.001
70-79	221	39.9	404	48.3	84	38.4		
≥80	54	9.7	148	17.7	87	39.7		
Estado civil								
Sin pareja	284	51.3	506	60.6	131	59.8		0.002
Con pareja	270	48.7	329	39.4	88	40.2	12.530	
Escolaridad (años)								
0	91	16.5	182	21.8	57	26.0	21.882	<0.001
1-4	278	50.3	441	52.8	119	54.3		
≥5	184	33.3	213	25.5	43	19.6		
Ingreso económico mensual (salario)**								
0	34	6.1	58	7.0	16	7.3	27.331	<0.001
<1	237	42.9	434	52.0	131	59.8		
2-3	212	38.3	268	32.1	60	27.4		
≥4	70	12.7	74	8.9	12	5.5		

*p <0.05;

**Sueldo mínimo del periodo de investigación: R$ 622.00.

**Tabla 2:** Características de salud de los adultos mayores de acuerdo con el estado de fragilidad. Uberaba, Minas Gerais, Brasil, 2012.

Variables	No frágil		Pre-frágil		Frágil		χ^2^	p*
	n	%	n	%	n	%		
Morbidilidades								
0	33	6.0	23	2.8	1	0.5	91.375	<0.001
1-4	303	54.7	325	38.9	58	26.5		
≥5	218	39.4	488	58.4	160	73.1		
Incapacidad funcional ABVD								
Si	55	9.9	185	22.1	90	41.1	96.323	<0.001
No	499	90.1	651	77.9	129	58.9		
Incapacidad funcional AIVD								
Si	278	50.2	572	68.4	202	92.2	129.756	<0.001
No	276	49.8	264	31.6	17	7.8		
Sintomas depresivos								
Si	86	15.5	216	25.8	116	53.0	114.458	<0.001
No	468	84.5	620	74.2	103	47.0		

*p<0.05;

ABVD: Actividades Básicas de la Vida Diaria;

AIVD: Actividades Instrumentales de la Vida Diaria.

Entre las morbilidades reportadas en ambos grupos, hubo prevalencia de hipertensión arterial, problemas de visión y columna, con mayor porcentaje en los categorizados como frágiles, seguido de los pre-frágiles y de los no frágiles.

En relación a la incapacidad funcional para las ABVD, las tasas de dependencia más altas fueron los que sufrían de incontinencia en los tres grupos.

Los adultos mayores frágiles tuvieron un mayor porcentaje de dependencia para transporte, tareas domésticas y compras comparado con aquellos categorizados como pre-frágiles y no frágiles.

Las variables incluidas en el modelo de regresión logística multivariada están presentadas en la Tabla 3. Los factores asociados con fragilidad y pre-fragilidad fueron las categorías del intervalo de la edad de 70-79 años y ≥80 años, una a cuatro morbilidades y cinco y más morbilidades, incapacidad funcional para las ABVD y las AIVD y presencia de sistemas depresivos. Por otro lado, la falta de una pareja, de ingresos económicos mensuales y el sexo femenino se asociaron a la pre-fragilidad (Tabla 3).

**Tabla 3:** Modelo final de regresión logística multinomial entre las categorías fragilidad y pre-fragilidad con las variables sociodemográficas y de salud. Uberaba, Minas Gerais, Brasil, 2012.

Variables	Pre-frágil			Frágil		
	OR	IC 95%	p*	OR	IC 95%	p*
Intervalo de edad (años)						
60-69	1			1		
70-79	1.68	1.31-2.16	0.001	2.13	1.40-3.31	0.001
≥80	2.19	1.50-3.29	0.001	7.40	4.40-12.46	0.001
Sexo						
Masculino	1			1		
Femenino	1.38	1.06-1.80	0.017	0.86	0.56-1.33	0.500
Estado civil						
Sin pareja	1.56	1.21-2.01	0.001	1.09	0.73-1.63	0.676
Con pareja	1			1		
Escolaridad (años)						
0	0.97	0.67-1.40	0.968	1.11	0.63-1.95	0.718
1-4	0.99	0.72-1.36	0.978	1.10	0.67-1.66	0.802
≥5	1			1		
Ingreso mensual (salario)†						
0	1.96	1.06-3.62	0.031	1.96	0.70-5.48	0.197
<1	1.34	0.88-2.03	0.173	1.77	0.83-3.77	0.139
2-3	1.03	0.68-1.55	0.888	1.31	0.61-2.82	0.483
≥4	1			1		
Morbilidades						
0	1			1		
1-4	1.41	0.79-2.53	0.249	4.28	0.52-35.08	0.176
≥5	2.43	1.34-4.41	0.003	9.16	1.12-74.89	0.039
Incapacidad funcional ABVD						
Si	1.87	1.33-2.63	0.001	3.06	1.98-4.73	0.001
No		1			1	
Incapacidad funcional AIVD						
Si	1.68	1.32-2.13	0.001	6.36	3.67-11.02	0.001
No	1			1		
Sintomas depresivos						
Si	1.44	1.07-1.94	0.016	4.05	2.72-6.02	0.001
No	1			1		

OR: *Odds Ratio*; IC: Intervalo de Confianza, *p <0.05;

1: Categoria de referencia;

† Sueldo mínino en 2012 in Brasil (R$ 622.00);

BADL: Actividades Básicas de la Vida Diária;

IADL: Actividades Instrumentales de la Vida Diária.

## Discusión

Investigaciones brasileñas ^6^^-^^8^ que abordaron el tema de fragilidad, encontraron prevalencias menores al ser comparados con el presente estudio, aunque en la ciudad de Santa Cruz - RN ^20^ fueron observadas altas prevalencia en los adultos mayores pre-frágiles (60.1%) y frágiles (17.1%). La variación de la prevalencia de fragilidad en estos estudios, puede estar relacionado al enfoque metodológico y los instrumentos de medida de los componentes del fenotipo de fragilidad y su influencia de las características socioeconómicas y de salud, además del contexto social de la población.

Resultados consistentes con este estudio se han verificado en la investigación de la Red FIBRA - Campinas-SP, cuyo mayor porcentaje de adultos mayores frágiles se produjo entre aquellos denominados los más viejos ^8^. Otra investigación realizada en Bahía verificó una asociación entre fragilidad y los grupos etarios; los categorizados más viejos, una alta probabilidad de fragilidad y pre-fragilidad (*p* <0.001) ^21^. Los adultos mayores considerados más viejos son más vulnerables a eventos estresantes, mayor sobrecarga funcional y dificultad para mantener la homeostasis ^3^, sugiriendo que cuanto más edad tiene el adulto mayor, aumenta la probabilidad de ser frágil ^20^^,^^22^.

Los adultos mayores frágiles tienen un alto riesgo de afectación en su salud y requieren de una mayor demanda de cuidados, lo que resulta en una sobrecarga tanto para las familias como para el sistema de salud. Por lo tanto, es importante que la familia se involucre en el cuidado de los adultos mayores frágiles, proporcionando un apoyo adecuado al adulto mayor. La inversión de medidas para prevenir la fragilidad asegura una mejor calidad de vida y menor costo para los servicios de salud. La demanda de atención a largo plazo y apoyo social refleja la necesidad de que estos adultos mayores sean el objetivo de las políticas públicas ^23^.

En relación al sexo, los resultados son corroborados por una estudio brasileño, que encontró que el sexo femenino era mas frecuentemente categorizado como pre-frágiles ^8^ siendo diferente de un estudio internacional ^24^ que no encontró asociación entre ambas variables. Este resultado puede estar relacionado con una pérdida mayor de la reserva fisiológica que sufren las mujeres comparada con el sexo masculino ^25^, además tienen mayores probabilidades de empeorar sus condiciones socioeconómicas y de salud, lo que puede reflejar una mayor posibilidad de que tengan mayor frecuencia de fragilidad ^26^. Además, las mujeres tienen una mayor expectativa de vida y menor índice de masa muscular ^27^, lo que puede favorecer la condición de pre-frágil al ser comparadas con los hombres.

Al respecto del estado civil, estudios realizados en el Brasil ^6^ corroboran los resultados del presente estudio, debido que hubo una mayor proporción de participantes sin pareja comparados con los no frágiles. Lo que concierne a los ingresos económicos, resultados divergentes fueron encontrados en un estudio realizado en Santa Cruz - RN ^20^, donde no se encontró una asociación entre los bajos ingresos económicos y la fragilidad en el adulto mayor que puede ser el resultado de desventajas acumuladas a lo largo de la vida, sumadas a los déficits relacionados con el proceso de envejecimiento ^8^.

En relación a las morbilidades, los resultados de este estudio confirmaron lo que se ha encontrado en diversos estudios ^2^^,^^3^^,^^9^^,^^10^^,^^20^. Al igual que en el presente estudio, la hipertensión arterial se destacó entre los adultos mayores de una comunidad taiwanesa, donde el 49.5% fueron frágiles y 47.7% pre-frágiles ^10^. Otro estudio determinó que los individuos con síndrome de fragilidad presentaron hipertensión arterial y otros riesgos cardiovasculares, HDL bajo e incremento de la circunferencia abdominal al ser comparados con los participantes no frágiles ^28^. La fragilidad puede aumentar el desarrollo o progresión de las enfermedades crónicas, probablemente a la disminución de la actividad o por mecanismos que interfieren en la homeostasis en el adulto mayor ^3^. De otro lado, la presencia de enfermedades concomitantes puede contribuir para el desarrollo de fragilidad en el adulto mayor ^29^, esa asociación fue identificada en un estudio realizado en Belo Horizonte, los autores encontraron que los adultos mayores frágiles tenían una alta probabilidad de presentar un alto número de comorbilidades (OR=1.27) ^6^.

En relación con las incapacidad funcional para las ABVD, el estudio mostro que el 70.7% de los adultos mayores sufrían de incontinencia urinaria (IU) presentaron al menos tres criterios indicativos de fragilidad y su ocurrencia fue mayor en los adultos mayores frágiles, lo que corrobora los hallazgos de la presente investigación ^30^. La IU en el adulto mayor frágil tiene una repercusión negativa, convirtiéndola en una condición que presenta varios factores de riesgo que interactúan, como los cambios inherentes a la edad y las comorbilidades ^31^, que sugiere la necesidad de investigaciones así como la implementación de acciones con la finalidad de reducir y prevenir esta incapacidad asociada con el síndrome de fragilidad. Entre las estrategias para minimizar la ocurrencia de IU, se destaca la orientación para los adultos mayores sobre la ingesta de alimentos, líquidos y prácticas que fortalecen los músculos del suelo pélvico. Las medidas tempranas para prevenir la IU y la condición de fragilidad en los adultos mayores pueden tener un impacto positivo en la vida de estos individuos, reduciendo la sobrecarga económica, psicológica y social ^30^.

Se observó asociación entre fragilidad y dependencia para la realización de las ABVD y las AIVD, corroborados por estudios brasileiros ^6^^,^^20^ y americanos ^2^^,^^3^. Un estudio realizado en Ribeirão Preto - SP mostró que cuanto mayor es el nivel de fragilidad, mayor es el nivel de dependencia en el adulto mayor ^12^. Por lo tanto, la dificultad para realizar actividades básicas e instrumentales de la vida diaria puede causar aumento de la dependencia y el surgimiento del síndrome de fragilidad ^32^.

En relación a las AIVD, investigando la asociación entre el apoyo social y la fragilidad se encontró una asociación significativa entre este síndrome y ayuda con las actividades domésticas ^9^, divergiendo del estudio actual que identificó la dependencia para las actividades domésticas, transporte y realizar compras. El síndrome de fragilidad está estrechamente relacionado con la discapacidad, requiriendo la incorporación de medidas preventivas para mitigar y disminuir la dependencia funcional en los adultos mayores, con la finalidad de promover un envejecimiento activo ^6^.

De forma similar a la presente investigación, en un estudio realizado con adultos mayores en la ciudad de Belo Horizonte-MG ^6^, la presencia de síntomas depresivos indicó una asociación con condiciones de fragilidad y pre-fragilidad (*p* <0.001). En la misma dirección, una revisión de la literatura identificó que síntomas depresivos como antecedentes que pueden favorecer el desarrollo del síndrome de fragilidad en el adulto mayor ^33^.

Conocer más profundamente la relación entre la fragilidad y la presencia de síntomas depresivos puede contribuir a aclarar los factores que influyen en su etiología y el pronóstico del síndrome, favoreciendo el desarrollo de acciones específicas y la integración de diferentes campos geriátricos ^34^.

Las limitaciones de este estudio están referentes al uso del cuestionario para el reporte de morbilidades y el tipo del diseño de estudio por ser transversal que dificulta el avance en el análisis del tiempo, debido al sesgo de causalidad, lo que no permite encontrar una relación causal.

## Conclusión

Los adultos mayores frágiles representaron el 13.6%, mientras que los adultos mayores pre-frágiles correspondieron al 52.0%. Los factores asociados a las categorías frágil y pre-frágil fueron, el intervalo de edad de 70-79 años y ≥80 años; una a cuatro morbilidades y cinco y más morbilidades; incapacidad funcional para las AIVD y ABVD y síntomas depresivos. Por otro lado, la falta de pareja, ingreso económico mensual y ser del sexo femenino se asociaron con la pre-fragilidad.

Estos resultados destacan la necesidad de acciones de salud enfocadas a las características socioeconómicas de los adultos mayores. Por lo tanto, se pueden establecer medidas preventivas considerando la mayor asociación del síndrome de fragilidad con aspectos socioeconómicos.

Además, la detección de fragilidad puede contribuir al establecimiento de medidas preventivas en su evolución. También existe la necesidad de monitorear las condiciones de salud de estos adultos mayores con el fin de posponer la aparición de comorbilidades, incluyendo depresión, y su control, con el fin de ayudar a favorecerlos para minimizar el desarrollo de este síndrome. Las actividades centradas en mantener la funcionalidad también pueden ayudar en este sentido.
